# AUTHOR'S RESPONSE

**DOI:** 10.4103/0019-5545.31601

**Published:** 2006

**Authors:** Amit Ranjan Basu

**Affiliations:** * Independent Researcher and Doctoral Candidate, Centre for Historical Studies, Jawaharlal Nehru University, New Delhi, India; *mailing address:* BE 318, Salt Lake, Kolkata 700064, West Bengal; e-mail: amitrbasu53@gmail.com

I am thankful to Professor Paul Hoff and Professor Shridhar Sharma for engaging with my ‘viewpoint’^1^ from different levels and offering their commentaries. This, including my response, I hope may enliven the debate on epistemological issues of Indian psychiatry.

Paul Hoff has appreciated the central issue of my argument that cultural and historical studies in psychiatry are crucial for the development of a critical knowledge. Particularly interesting is his observation on the politics of knowledge pointing out the prejudiced historical narratives that *only* saw the history of psychiatry in India as the successful western concepts replacing irrational, superstitious and non-scientific practices. Hoff's critique is with my (intended) ‘omission’ of *enlightenment project* and being ‘too enthusiastic’ about Foucault's work on psychiatry (Sharma too shared this view), which may lead us to the danger of relativism.

First, I never attempted to *reject* or *omit* the ‘enlightenment project’. I argued that our ‘enlightenment’ with European modernity has come through colonialism. Collaborative historians had established a theory of *Bengal Renaissance* until it was thoroughly criticized in the recent past by eminent Indian social scientists. Colonialism being the entry point, the discourse of rationality and reason emerging from the *enlightenment* had a very different implication in practice of our modernity.^2^ The power equations operating through a modern knowledge system were different and as post-colonial subjects, our modern knowledge is always *deferred* and is in a perpetual *lack.* Without criticizing *enlightenment,* I think it is not possible to see other alternative resources available here. Without carefully locating the fracture lines of the dominant, linear, progessivist narratives of the *enlightenment,* one cannot find *other reasons* that are valid in a non-western context. The power of *enlightenment* reason appropriates the multitude of reasons for global, homogeneous categories. This, I am convinced still needs to be pursued, which can be done by taking a relativistic position first. It is the critique (not rejection) of *enlightenment* that has given birth to contem-porary post-colonial theories that are seriously questioning the given modernity not only in South Asia but also elsewhere.

The influence of Foucault's work on my essay starts with the appreciation that he has changed the history of psychiatry by his intervention and, if one is touching on theoretical issues, it is difficult to avoid Foucault (whether one agrees with him or not) in contemporary historiography of psychiatry. Foucault provided us with effective tools to explore the play of power/knowledge, which would scientifically convince us to exclude certain population groups that do not conform to the concept of modern governance. However, this did not make Foucault to claim a general theory of power and, in my observation, I have seen his shortcomings of understanding colonial power.^3^,^4^

I also think it is wrong to dissolve the distinction between Foucault's theory and the *Antipsychiatry* movement. While all antipsychiatric writings were essentially anti-state and relied on Marxism, existentialism and left-liberalism, Foucault instead conceptualized (psychiatric) power differently and did not follow an oppositional binary paradigm.^5^ His critique was not targeted towards state or any oppressive group, rather towards the way madness has been conceived and described by a modern human science. However, the ideologues of *Antipsychiatry* movement R.D. Laing and David Cooper have claimed Foucault's work to be similar to theirs; but on a closer reading, it is not difficult to see that both are different in their philosophical positions. Yet I think Hoff's feeling of reading my text as having the tendency to focus on the critique of *enlightenment* is somewhat justified beause he read an abridged version of my original article.

Shridhar Sharma's comments on history, I find problematic. His problem, I guess, is generated from his mis-readings of *historicism.* I acknowledge this partly to be my fault as I did not explain the term ‘historicism’ exhaustively, which may have appeared difficult to those readers who are not familiar with contemporary social science discourse. He has not discussed historical methods nor could he appreciate that I was making a critique of the dominant narratives that are representing history of Indian psychiatry in a particular way, which appears to be the only valid fact.

I have challenged this with a contemporary review and some samples from a corpus of colonial and post-colonial archival materials to show that Indian psychiatry mimicked Euro-American models uncritically because it has hardly looked at its cultural resources seriously to develop its own conceptual repertoire. A forthcoming critical review has eruditely argued this with more references.^6^ In addition, dichotomous analyses can be a trap between traditional/modern conflicts. Glorifying the tradition only becomes possible through the grid of modern. I think it would be wrong to assume that non-western psychiatric practices did not change with the massive changes in culture and polity. Similarly *Ayurveda* is not the only medical system representing India; in fact, there are many. Somehow, Ayurveda and India are simplistically made synonymous in many historical writings!

It is good to see that Sharma has acknowledged that ‘to understand the development of mental hospitals, it is relevant to know the political developments in India during that time’. However, among the studies he has referred, James Mills' study goes more in favour of the approach I have taken. It is quite clear from Mills' commentary that he did not want to write a positivist history.

I conclude by saying that Hoff's acknowledgement of the problem is encouraging as it opens up the dialogue and has an interest to engage with what he calls ‘locally-based research’. Here my difference with him has the potential to become productive. Sharma, on the other hand, appears less informed about contemporary interdisciplinary history and fails to offer any space for critical engagement or any possibilities out of his critique.
